# RGN-259 (thymosin β4) improves clinically important dry eye efficacies in comparison with prescription drugs in a dry eye model

**DOI:** 10.1038/s41598-018-28861-5

**Published:** 2018-07-12

**Authors:** Chae Eun Kim, Hynda K. Kleinman, Gabriel Sosne, George W Ousler, Kyeongsoon Kim, Sinwook Kang, Jaewook Yang

**Affiliations:** 1 Department of Ophthalmology, Inje University College of Medicine, Inje University Busan Paik Hospital, Busan, 47392 Korea; 2 Department of Biochemistry and Molecular Biology, The George Washington University School of Medicine, Washington D.C, USA; 3 Departments of Ophthalmology and Anatomy/Cell Biology, Kresge Eye Institute, Wayne State University School of Medicine, Detroit, Michigan USA; 4 Ora. Inc., 300 Brickstone Square, Andover, MA USA; 5 Department of Pharmaceutical Engineering, Inje University, Gimhae, Republic of Korea; 6 ReGenTree, LLC, 116 Village Boulevard, Suite 200, Princeton, NJ USA; 7 T2B infrastructure Center for Ocular Disease, Inje University Busan Paik Hospital, Busan, 47392 Korea

## Abstract

This study evaluated the clinical activity of RGN-259 (thymosin β4) in comparison with cyclosporine A (CsA), diquafosol (DQS), and lifitegrast (LFA) in a murine model of dry eye. The model was NOD.B10-*H2*^*b*^ mice in a 30–40% humidified environment together with daily scopolamine hydrobromide injections for 10 days. After desiccation stress, all drugs were evaluated after 10 treatment days. RGN-259 increased tear production similar to that in the DQS- and LFA-treated mice while CsA was inactive. RGN-259 improved corneal smoothness and decreased fluorescein staining similar to that of LFA group while CsA and DQS were inactive. Corneal epithelial detachment was reduced by RGN-259, and DQS and LFA showed similar activity but the CsA was inactive. RGN-259 increased conjunctival goblet cells and mucin production comparable to that seen with CsA, while DQS and LFA were inactive. RGN-259 reduced the over-expression of inflammatory factors comparable to that with CsA and LFA, while DQS was inactive. RGN-259 increased mucin production comparable to that observed with CsA, while DQS and LFA were inactive. In conclusion, RGN-259 promoted recovery of mucins and goblet cells, improved corneal integrity, and reduced inflammation in a dry eye mouse model and was equal to or more effective than prescription treatments.

## Introduction

Dry eye is a chronic ocular surface disease, and can cause visual morbidity that affects the quality of life^1^. In 2017, the TFOS Dry Eye Workshop II redefined dry eye as “a multifactorial disease of the ocular surface characterized by a loss of homeostasis of the tear film, and accompanied by ocular symptoms, in which tear film instability and hyperosmolarity, ocular surface inflammation and damage, and neurosensory abnormalities play etiological roles”^2^. The tear film is composed of a lipid layer, an aqueous layer, and a mucous layer^1–7^. Persistent dry eye leads to an imbalance in the tear film resulting in damage to the corneal epithelial cells and loss of conjunctival goblet cells^1,7–9^.

Prolonged dry eye is associated with increases in the levels of inflammatory cytokines in both the conjunctiva and tears^10,11^. It also increases the numbers of T lymphocytes in the conjunctiva and the expression of adhesion molecules in the conjunctival epithelium^10–13^. Long-term dry eye is associated with increased expression of inflammatory factors, such as tumor necrosis factor-alpha (TNF-α), matrix metalloproteinase (MMP-2, MMP-9), intercellular adhesion molecule-1 (ICAM-1), and vascular cell adhesion molecule-1 (VCAM-1) in the ocular surface^7,8,14–16^.

Mucins are large glycoproteins that are the major components of mucous secretions^17^. On the ocular surface, mucins act as a lubricant in both the cornea and conjunctiva, and are known to act as a physical barrier to stabilize the tear film and to protect the eye from outside pathogens^17^. Mucins are categorized into cell surface-associated mucins (Muc1/4/16) and gel-forming mucins (Muc5AC), which are secreted by the corneal and conjunctival epithelium^18–20^. However, these mucins have also been reported to be secreted in response to inflammation^21^. Mucin expression is down-regulated by pro-inflammatory cytokines in a conjunctival epithelial cell line^22,23^. In addition, mucin expression is down-regulated in patients with dry eye and in dry eye animal models^24–27^. Recent studies identified the anti-inflammatory effects of mucins in the gastrointestinal tract and in the ocular conjunctiva^28,29^.

Thymosin beta 4 (Tβ4) is a 43-amino acid G-actin binding protein found in almost all cells, including platelets, macrophages, and polymorphonuclear cells^30–33^. It is not present in red blood cells. Tβ4 promotes wound repair and regeneration in the skin, eye, heart, and nervous system in various animal models^34–41^. Tβ4, which is also known to modulate the expression of multiple signaling molecules, plays a role in the down-regulation of transcription factors for inflammatory chemokines, cytokines, and metalloproteinases^30,32,42^. Tβ4 promotes ocular surface healing, increased corneal epithelial cell migration, and decreased corneal pro-inflammatory cytokine levels in multiple models of corneal injury *in vivo*. Recent clinical studies have shown that topical 0.1% Tβ4 (RGN-259) may be helpful in the treatment of neurotrophic keratitis^43^. In a phase II clinical trial, topical 0.1% Tβ4 significantly improved the signs and symptoms of patients with dry eye, and no side effects were observed^44,45^. Tβ4 also showed efficacy for improving corneal healing in a mouse model of dry eye, but several additional parameters, including mucin and tear production were not evaluated. Furthermore, the efficacy of RGN-259 in a head-to-head comparison with that of already approved dry eye treatments has not been evaluated.

In this study, we investigated the effects of RGN-259 topical treatment on the cornea, conjunctiva, and lacrimal glands in a murine dry eye model, and we also compared the effects of RGN-259 with that of prescription drugs. Specifically, we investigated the changes in tear production, the corneal irregularity score, and corneal fluorescein staining on the ocular surface. The histological parameters examined were corneal epithelial cell detachment, conjunctival goblet cell number, and the expression of inflammatory factors and mucins in the cornea, conjunctiva, and lacrimal glands.

## Results

### RGN-259 increased tear production

**Figure 1:**
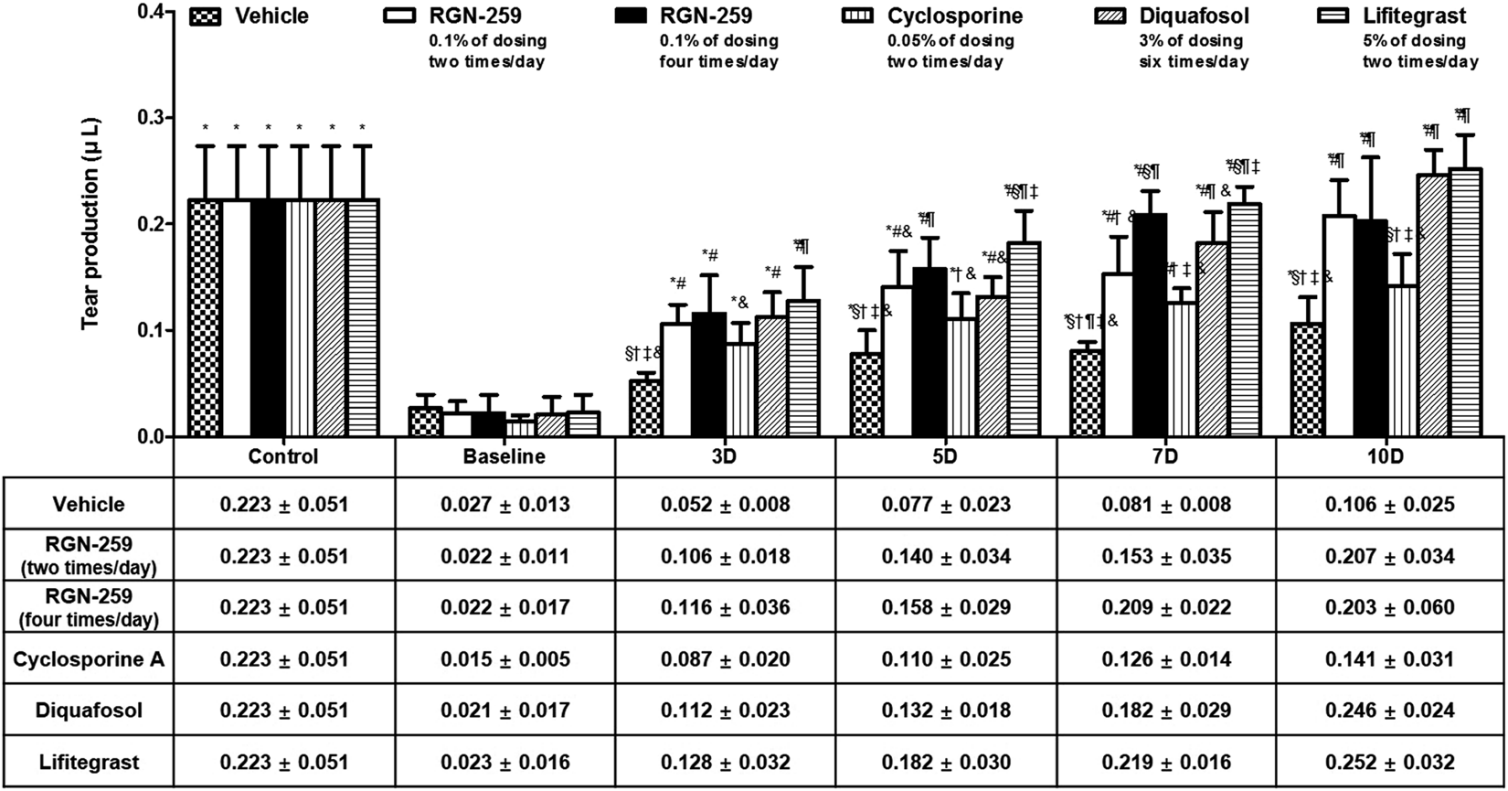
RGN-259 increased tear production comparable to that of DQS and LFA. Changes in tear volume in the experimental dry eye model are shown. ^*^P < 0.05 vs. the corresponding value in the baseline desiccation stressed group. ^*#*^*P* < 0.05 vs. the value in the vehicle-treated group. ^*§*^*P* < 0.05 vs. the value in the RGN-259, 2 times per day group. ^*†*^*P* < 0.05 vs. the value in the RGN-259, 4 times/day group. ^*¶*^*P* < 0.05 vs. the value in the CsA group. ^*‡*^*P* < 0.05 vs. the value in the DQS group. ^*&*^*P* < 0.05 vs. the value in the LFA group.

All of the treated mice and the vehicle control group showed increased tear production with time over the baseline values of desiccation stress at day 0 of treatment. Mice receiving RGN-259 2 times per day exhibited a 9.3-fold increase after 10 days compared with that of the desiccation stress baseline after 10 days of inducing dry eye (0.207 ± 0.034 μL vs 0.022 ± 0.011 μL) (*P* < 0.05) (Fig. 1). Whereas, the group that received RGN-259 4 times per day exhibited a 9.1-fold increase after 10 days compared with that of the desiccation stress baseline group (0.203 ± 0.060 μL vs 0.022 ± 0.017 μL) (*P* < 0.05). The CsA, DQS, and LFA groups exhibited 9.5-, 11.9-, and 11.1-fold increases after 10 days of treatment (0.141 ± 0.031, 0.246 ± 0.024, and 0.252 ± 0.032 μL), respectively, compared with the desication stress baseline values (0.015 ± 0.005, 0.021 ± 0.017, and 0.023 ± 0.016 μL) (*P* < 0.05). Importantly, the RGN-259 treated mice (both 2 and 4 times per day) showed a 2.0-fold and 1.9-fold increase in tear volume relative to that of the vehicle-treated group after 10 days of treatment (0.207 ± 0.034, 0.203 ± 0.060, vs 0.106 ± 0.025 μL, in the 2 and 4 times/day RGN-259, and vehicle treated groups, respectively). Both RGN-259-treated groups showed increased tear volumes comparable to those seen in the DQS and LFA groups (0.246 ± 0.025, and 0.252 ± 0.032 μL), and there was no significant difference in the tear volumes between the two RGN-259-treated groups and the DQS and LFA groups. It should also be noted that increases in the tear volume by RGN-259, DQS, and LFA over that of the control vehicle were seen as early as day 3 of treatment. The tear volume after CsA treatment was similar to that of the vehicle control at 10 days of treatment, and therefore CsA was inactive for increasing tear production after 10 days of treatment.

### RGN-259 improved corneal smoothness

**Figure 2:**
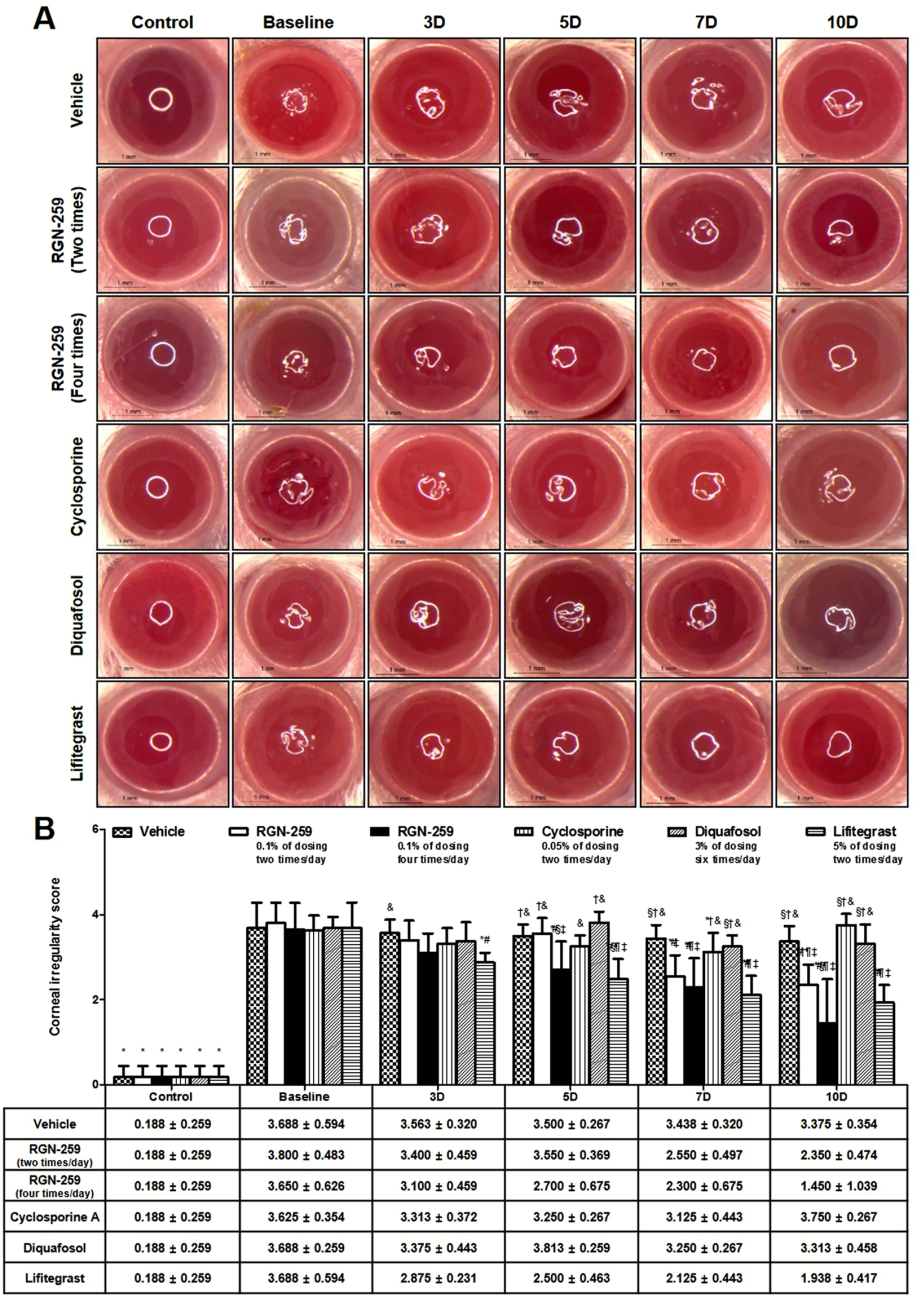
RGN-259 reduced corneal surface irregularities comparable to that of LFA. (**A**) Corneal smoothness was imaged using a microscope. Scale bar = 1 mm. (**B**) Change in corneal irregularity scores. ^*^P < 0.05 vs. the corresponding value in the baseline desiccation stressed group. ^*#*^*P* < 0.05 vs. the value in the vehicle-treated group. ^*§*^*P* < 0.05 vs. the value in the RGN-259, 2 times per day group. ^*†*^*P* < 0.05 vs. the value in the RGN-259, 4 times per day group. ^*¶*^*P* < 0.05 vs. the value in the CsA group. ^*‡*^*P* < 0.05 vs. the value in the DQS group. ^*&*^*P* < 0.05 vs. the value in the LFA group.

The corneal irregularity score of the baseline after 10 days of desiccation stress exhibited a large increase when compared with the untreated normal eyes (Fig. 2A). After 10 days of drug treatment, the vehicle, CsA, and DQS groups did not show improvement in the distortion of the white ring over the baseline observed after induction of dry eye. Thus, CsA and DQS were considered inactive for promoting corneal smoothness. In contrast, RGN-259 at 2 and 4 times per day and LFA showed improvement in the smoothness of the white ring over the vehicle alone treatment at 10 days of drug treatment. The corneal irregularity scores in the groups treated with RGN-259 at 2 and 4 times per day decreased in a dose-dependent manner to 38.2% and 60.3% at 10 days, respectively, when compared with the vehicle control group (2.350 ± 0.474, 1.450 ± 1.039 vs and 3.375 ± 0.354 score, in the 2 and 4 times/day RGN-259, and vehicle treated groups, respectively) (*P* < 0.05) (Fig. 2B). The decrease in the corneal irregularity scores for the RGN-259 treated groups was comparable to the decrease seen in the LFA group (47.5%), and showed activity by 7 days of treatment.

### RGN-259 increased healing as shown by reduced corneal fluorescein staining

**Figure 3:**
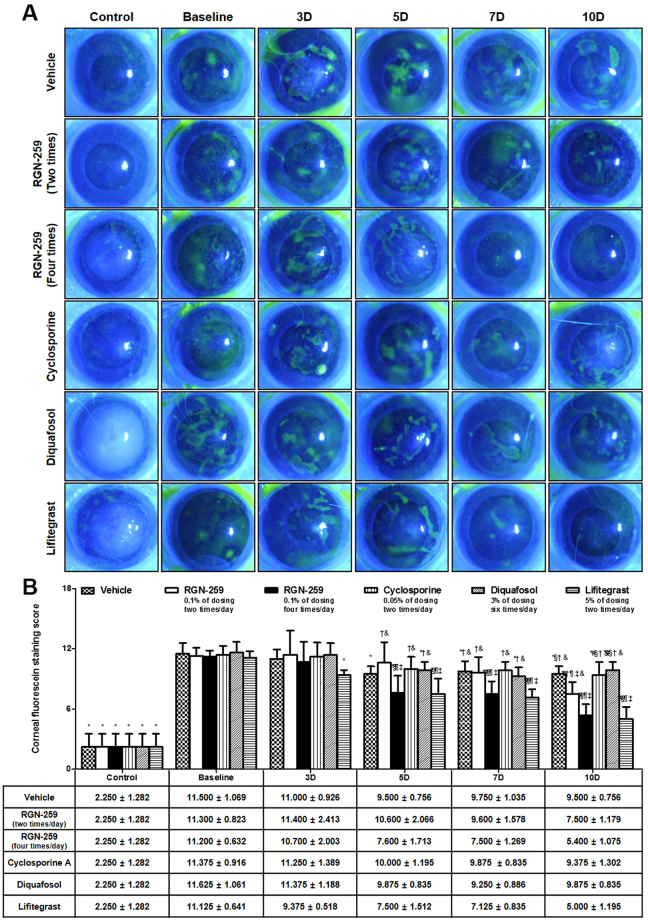
RGN-259 reduced corneal fluorescein staining comparable to that of LFA. (**A**) Corneal fluorescein staining was imaged using a slit lamp biomicroscope. (**B**) Change in the corneal fluorescein staining scores. ^*^P < 0.05 vs. the corresponding value in the baseline desiccation stressed group. ^*#*^*P* < 0.05 vs. the value in the vehicle-treated group. ^*§*^*P* < 0.05 vs. the value in the RGN-259, 2 times per day group. ^*†*^*P* < 0.05 vs. the value in the RGN-259, 4 times per day group. ^*¶*^*P* < 0.05 vs. the value in the CsA group. ^*‡*^*P* < 0.05 vs. the value in the DQS group. ^*&*^*P* < 0.05 vs. the value in the LFA group.

As shown in Fig. 3A, the control corneas from a normal healthy murine eye did not show any uptake of the fluorescent dye, indicating an intact epithelial barrier. However, the baseline dry eye desiccation stress-induced corneas showed a patchy staining pattern, indicating a damaged corneal epithelial barrier. The corneal fluorescein staining score of the baseline desiccation stress group before treatment (11.354 was the average score), exhibited approximately a 5-fold increase, compared with the control normal group (2.250 ± 1.282 score) (*P* < 0.05) (Fig. 3B). Damage to the corneal epithelial barrier in both the RGN-259 (2 and 4 times per day) and the LFA groups after 10 days of treatment decreased when compared with the vehicle-treated group. When compared with the baseline desiccation stress-induced group, the groups treated with DQS, vehicle, CsA, RGN-259 (2 times/day), RGN-259 (4 times per day), and LFA showed a 15.1%, 17.4%, 17.6%, 33.6%, 51.8%, and 55.1% decrease, respectively in the corneal fluorescein staining scores at 10 days. The group that received RGN-259 4 times per day showed a significant decrease in corneal fluorescein staining compared with the group that received RGN-259 2 times per day (5.400 ± 1.075 vs 7.500 ± 1.179 score in the RGN-259 4 times and 2 times per day groups, respectively), and both RGN-259 groups showed a decrease comparable to that seen in the LFA group (5.000 ± 1.195 score). The DQS- and CsA-treated corneas were similar to the vehicle-treated control and were considered not active for reducing corneal fluorescein staining.

### RGN-259 reduced epithelial cell detachment in the cornea

**Figure 4:**
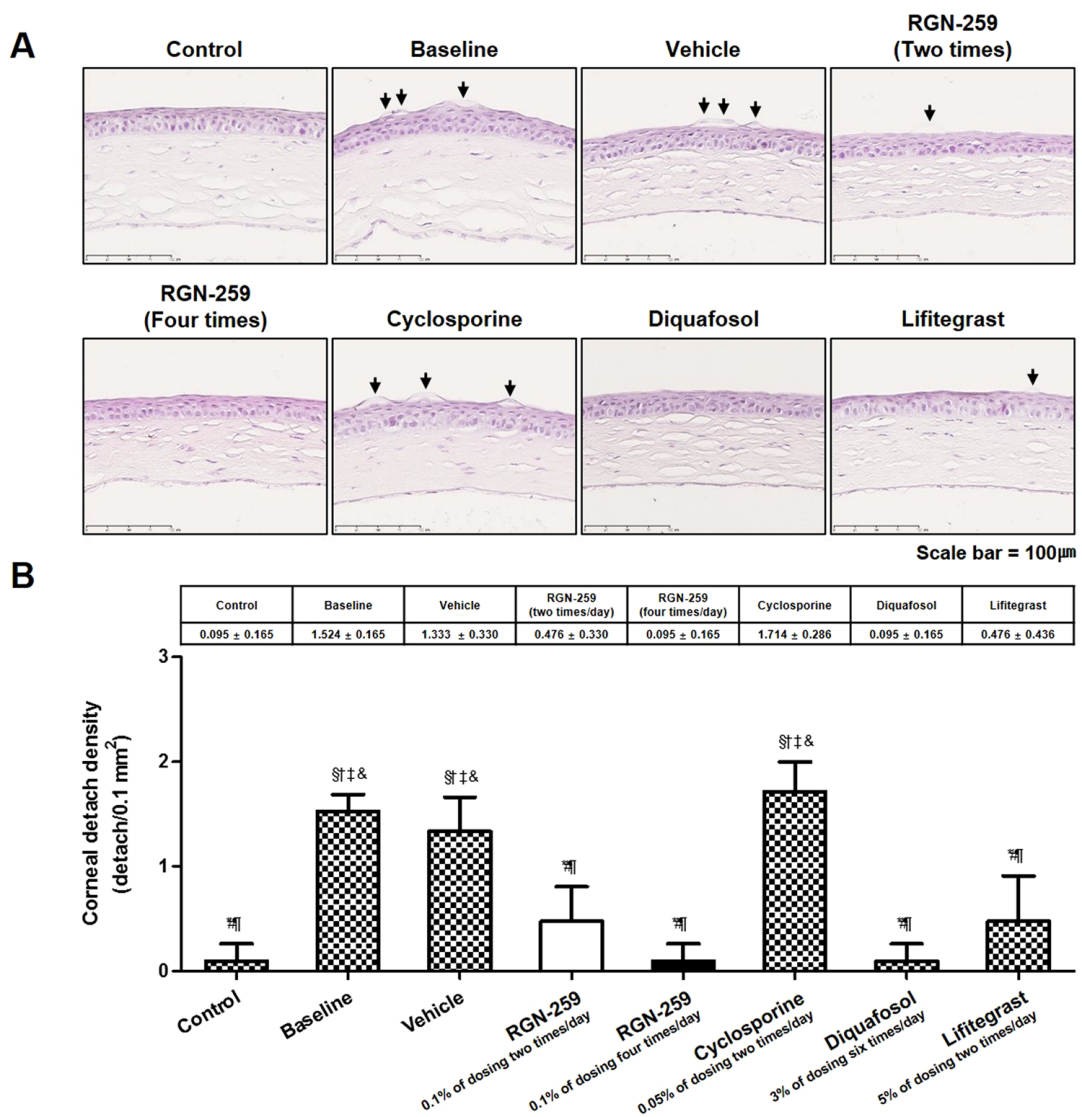
RGN-259 reduced detachment of corneal epithelial cells comparable to that of DQS. (**A**) The sections are stained with hematoxylin-eosin. The arrows indicate the detached corneal epithelial cells. Scale bar = 100 μm. (**B**) The number of detached corneal epithelial cell. ^*^P < 0.05 vs. the corresponding value in the baseline desiccation stressed group. ^*#*^*P* < 0.05 vs. the value in the vehicle-treated group. ^*§*^*P* < 0.05 vs. the value in the RGN-259, 2 times per day group. ^*†*^*P* < 0.05 vs. the value in the RGN-259, 4 times per day group. ^*¶*^*P* < 0.05 vs. the value in the CsA group. ^*‡*^*P* < 0.05 vs. the value in the DQS group. ^*&*^*P* < 0.05 vs. the value in the LFA group.

The corneas of the NOD.B10.*H2*^*b*^ mice before and after various treatments were stained with hematoxylin-eosin (HE) (Fig. 4A). The number of detached corneal epithelial cells was increased by 16-fold in the baseline desiccation stress group when compared with the control healthy group (1.524 ± 0.165 cells/0.1 mm^2^ vs. 0.095 ± 0.165 cells/0.1 mm^2^, *P* < 0.05) (Fig. 4B). When compared with the baseline group, the DQS and LFA groups showed a 93.8% (0.095 ± 0.165 cells/0.1 mm^2^) and 68.8% (0.476 ± 0.436 cells/0.1 mm^2^) decrease, respectively in the number of detached corneal epithelial cells (*P* < 0.05). However, the CsA group was inactive in decreasing the detachment of corneal epithelial cells (1.714 ± 0.286 cells/0.1 mm^2^). When compared with the baseline desiccation stress group, RGN-259 at 2 and 4 times per day showed a 68.8% and 93.8% decrease, respectively, in the number of detached corneal epithelial cells (*P* < 0.05). Furthermore, when compared to the vehicle- treated control, the two RGN-259 treated groups showed a statistically significant difference (0.476 ± 0.330, 0.095 ± 0.165, and 1.333 ± 0.330 cells/0.1 mm^2^, *p* < 0.05 in the 2 and 4 times/day RGN-259, and vehicle treated groups, respectively), and the decrease was dose-dependent for reducing corneal epithelial cell detachment. However, there were no significant differences, and the activity of the two RGN-259 groups was comparable to that of the DQS and LFA activity.

### RGN-259 regenerated goblet cells

**Figure 5:**
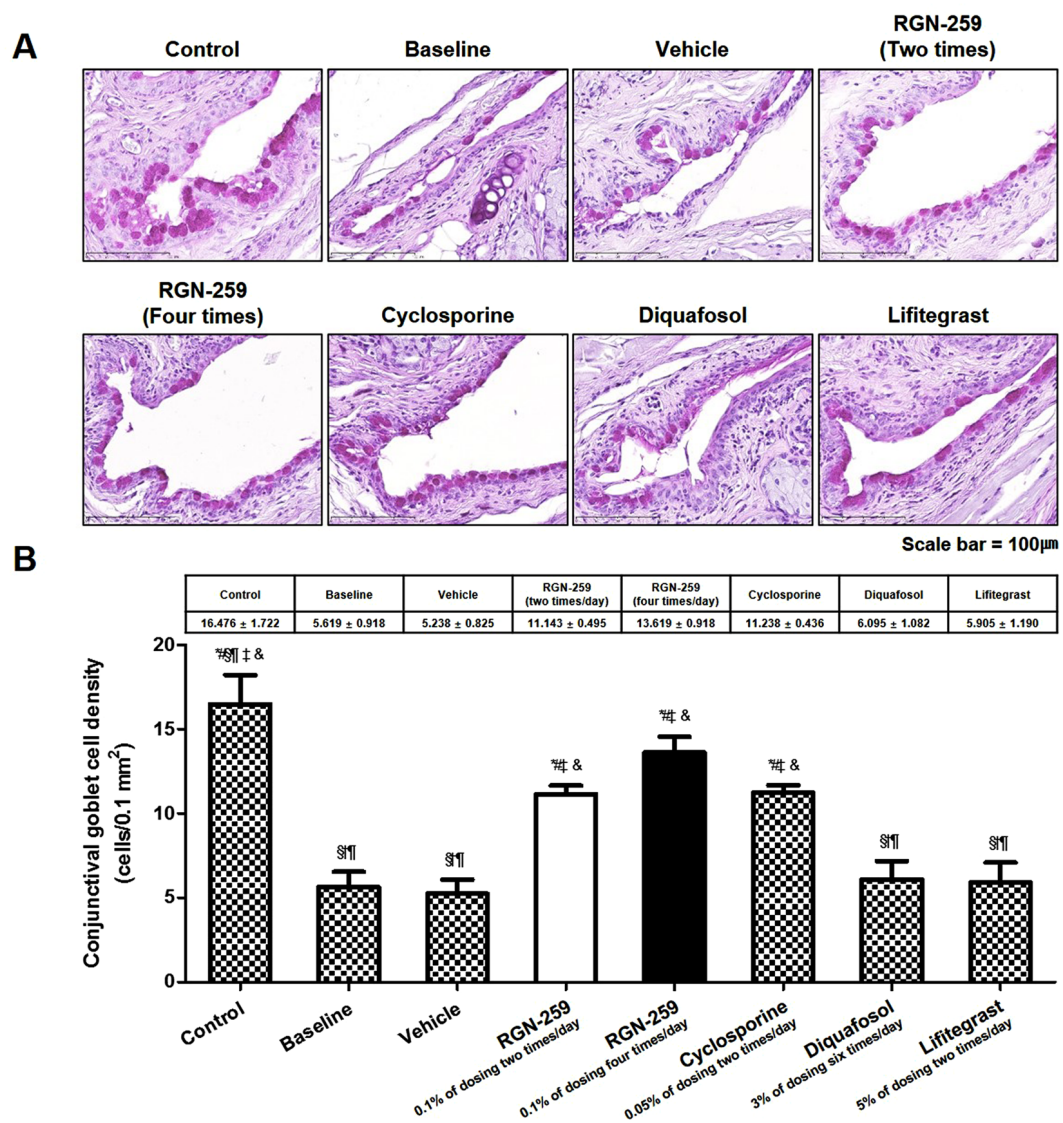
RGN-259 increased conjunctival goblet cell densities comparable to that of CsA. (**A**) Periodic acid Schiff staining. The strong violet color indicates the conjunctival goblet cells. Scale bar = 100 μm. (**B**) The number of conjunctival goblet cells. ^*^P < 0.05 vs. the corresponding value in the baseline desiccation stressed group. ^*#*^*P* < 0.05 vs. the value in the vehicle-treated group. ^*§*^*P* < 0.05 vs. the value in the RGN-259, 2 times per day group. ^*†*^*P* < 0.05 vs. the value in the RGN-259, 4 times per day group. ^*¶*^*P* < 0.05 vs. the value in the CsA group. ^*‡*^*P* < 0.05 vs. the value in the DQS group. ^*&*^*P* < 0.05 vs. the value in the LFA group.

Compared with the unstressed normal control mice, the desiccation stress-induced group showed a 65.9% decrease in the number of conjunctival goblet cells (16.476 ± 1.722 vs. 5.619 ± 0.918 cells/0.1 mm^2^) (Fig. 5). After 10 days of vehicle treatment, the number of goblet cells did not increase when compared to the baseline before treatment. Compared with the baseline desiccation stress-induced group, the groups treated 2 and 4 times per day with RGN-259, showed a 2.0- and 2.4-fold increase (11.143 ± 0.495 and 13.619 ± 0.918 cells/0.1 mm^2^, *P* < 0.05), respectively in the number of conjunctival goblet cells. The number of conjunctival goblet cells increased by 2.0-, 1.1-, and 1.1-fold in the CsA, DQS, and LFA groups (11.238 ± 0.436, 6.095 ± 1.082, and 5.905 ± 1.190 cells/0.1 mm^2^), respectively, when compared with baseline the desiccation stress group indicating that the DQS and LFA were inactive in increasing goblet cell numbers. In the group treated 4 four timer day with RGN-259, the conjunctival goblet cells recovered to levels comparable to that seen in the normal unstressed control mice. The number of conjunctival goblet cells in the two RGN-259 treated groups recovered to levels comparable to those seen in the CsA group. No significant differences were observed between the activity in both the RGN-259 and the CsA group.

### RGN-259 induced mucin production in the conjunctiva

**Figure 6:**
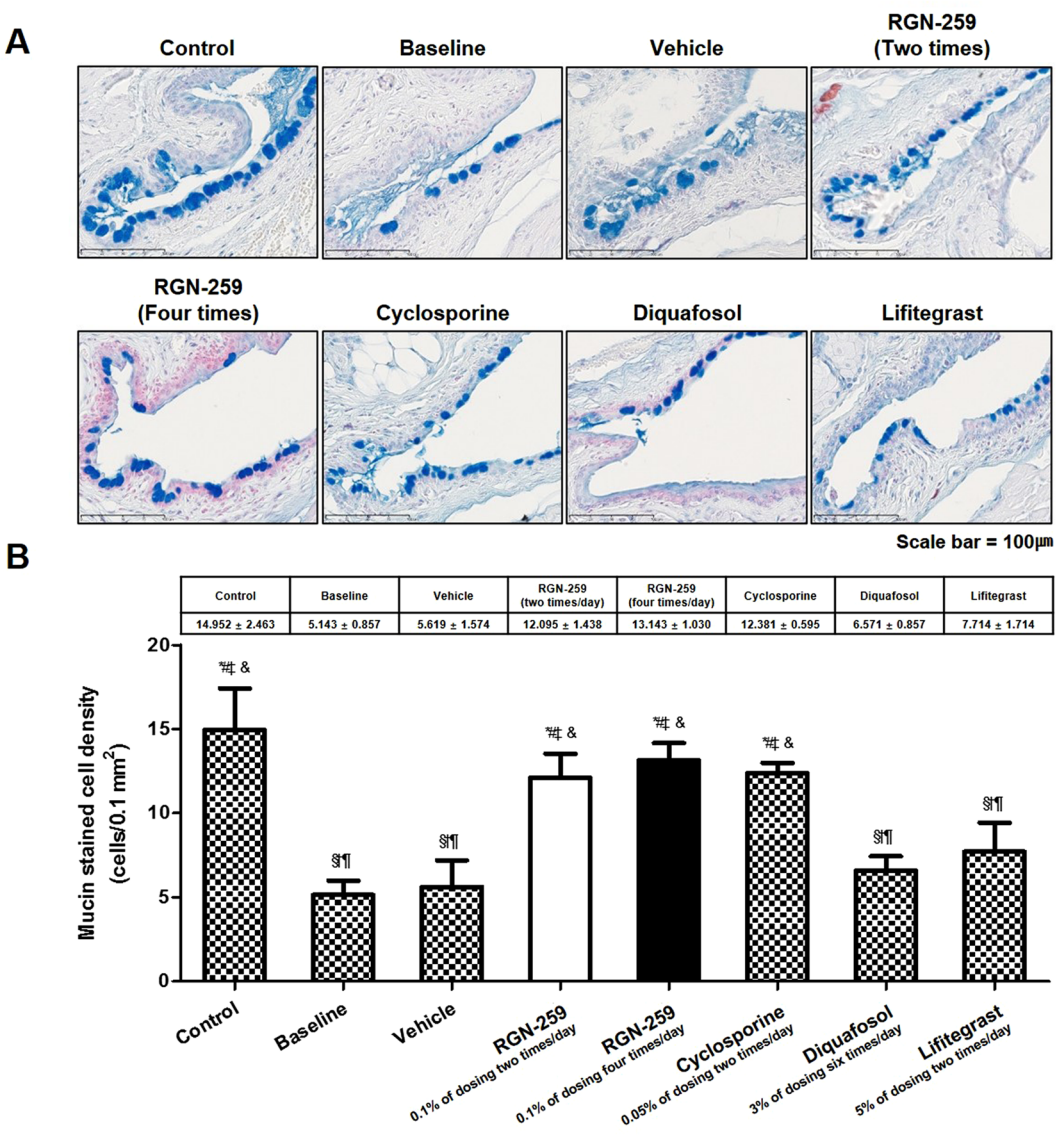
RGN-259 increased conjunctival mucin staining comparable to that of CsA. (**A**) Mucin staining. The strong blue color indicates the conjunctival mucin. Scale bar = 100 μm. (**B**) Quantitation of conjunctival mucin. ^*^P < 0.05 vs. the corresponding value in the baseline desiccation stressed group. ^*#*^*P* < 0.05 vs. the value in the vehicle-treated group. ^*§*^*P* < 0.05 vs. the value in the RGN-259, 2 times per day group. ^*†*^*P* < 0.05 vs. the value in the RGN-259, 4 times per day group. ^*¶*^*P* < 0.05 vs. the value in the CsA group. ^*‡*^*P* < 0.05 vs. the value in the DQSl group. ^*&*^*P* < 0.05 vs. the value in the LFA group.

The mucin of the conjunctiva was stained with alcian blue (Fig. 6A). The desiccation-induced baseline group showed a 65.6% decrease in mucin relative to the normal healthy controls (5.143 ± 0.857 cells/0.1 mm^2^ vs 14.952 ± 2.463 cells/0.1 mm^2^, *P* < 0.05) (Fig. 6B). When compared with the desiccation-stressed baseline group before treatment, the mucin staining in the conjunctiva increased by 2.4-, 2.6-, and 2.4-fold in the groups treated 2 and 4 times per day with RGN-259 and with CsA (12.095 ± 1.438, 13.143 ± 1.030, and 12.381 ± 0.595 cells/0.1 mm^2^, *P* < 0.05, respectively). The mucin staining in the conjunctiva increased by 1.1-, 1.3-, and 1.5-fold in the vehicle-, DQS-, and LFA- treated groups (5.619 ± 1.574, 6.571 ± 0.857, and 7.14 ± 1.714 cells/0.1 mm^2^), respectively, when compared with the baseline group, indicating that DQS was inactive and the LFA trended toward weak activity. The mucin staining in the two RGN-259-treated groups and in the CsA group increased significantly to levels seen in the unstressed control mice. No significant difference was seen in the staining between the two RGN-259 treated groups and the CsA group.

### RGN-259 reduced inflammatory factors in the lacrimal glands

**Figure 7:**
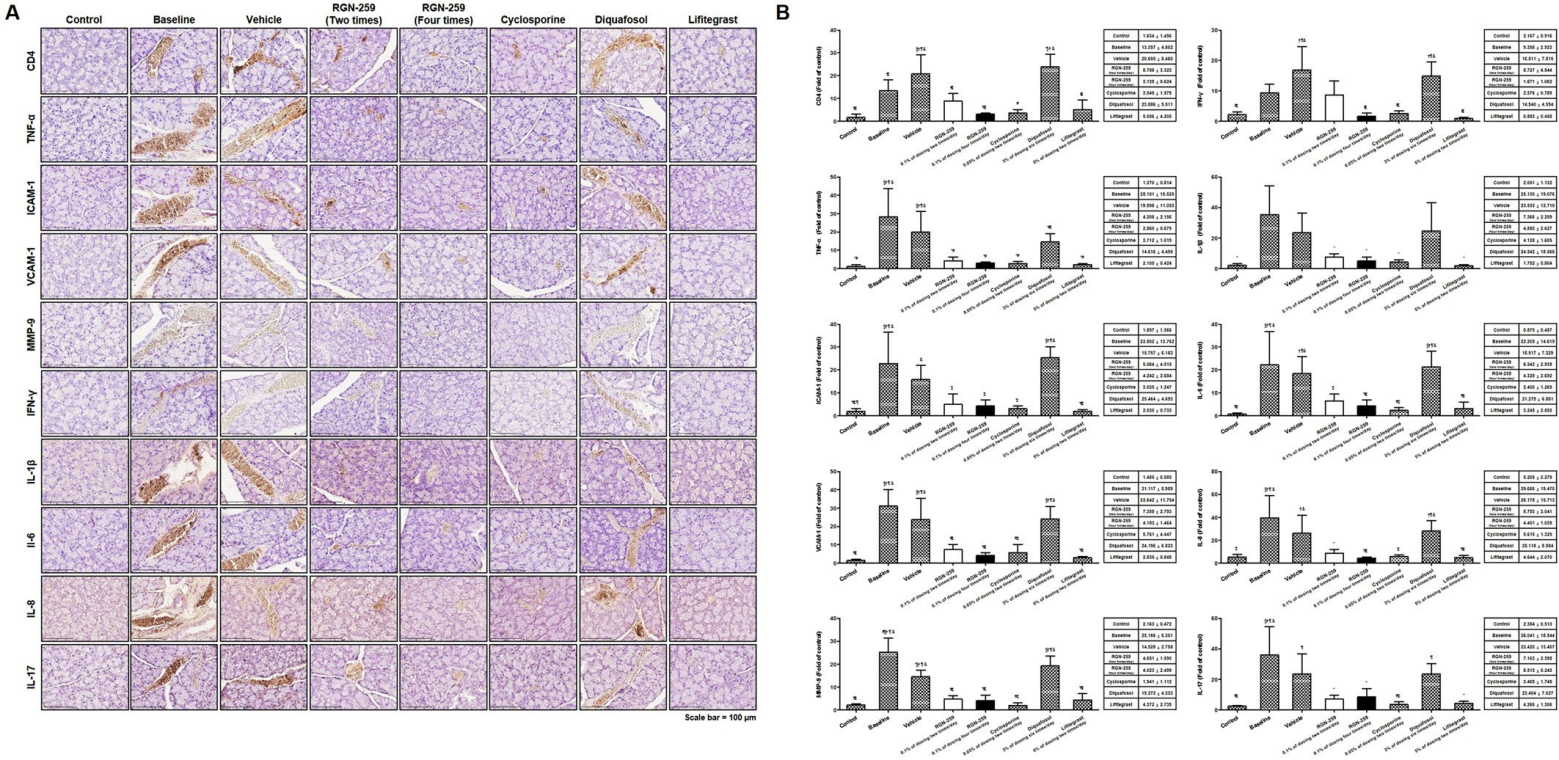
RGN-259 reduced the expression of inflammatory factors comparable to that of CsA and LFA. (**A**) Immunohistochemistry for inflammatory factors in the lacrimal glands of mice. Scale bar = 100 μm. (**B**) The quantitative expression of inflammatory factors. ^*^P < 0.05 vs. the corresponding value in the baseline desiccation stressed group. ^*#*^*P* < 0.05 vs. the value in the vehicle-treated group. ^*§*^*P* < 0.05 vs. the value in the RGN-259, 2 times per day group. ^*†*^*P* < 0.05 vs. the value in the RGN-259, 4 times per day group. ^*¶*^*P* < 0.05 vs. the value in the CsA group. ^*‡*^*P* < 0.05 vs. the value in the DQSl group. ^*&*^*P* < 0.05 vs. the value in the LFA group.

The immunostaining for cluster of differentiation 4 (CD4), tumor necrosis factor-alpha (TNF-α), intracellular adhesion molecule 1 (ICAM-1), vascular cell adhesion molecule 1 (VCAM-1), MMP-9, interferon (IFN)-γ, interleukin (IL)-1β, IL-6, IL-8, and IL-17. CD4, a glycoprotein on the surface of immune cells, was significantly increased in the lacrimal glands of mice subjected to desiccation stress (8.2-fold increase over the control normal group, *P* < 0.05) (Fig. 7). Compared with the baseline desiccation stress-induced group, groups treated 2 and 4 times per day with RGN-259 suppressed CD4 expression by 34.4% and 76.6% (5.4- and 1.9-fold increase over the control normal group) (*P* < 0.05). Compared with the desiccation stress baseline group, the CsA and LFA groups suppressed CD4 expression by 73.5% (2.2-fold increase over the control normal group) and 62.3% (3.1-fold increase over the control normal group, *P* < 0.05). However, no decrease in the CD4 expression was observed in the DQS group (14.6-fold increase over the control normal group). Mediators of inflammation, including TNF-α (22.1-fold increase over the control normal group), ICAM-1 (12-fold over the control normal group), VCAM-1 (21-fold increase over the control normal group), and MMP-9 (11.6-fold increase over the control normal group) were all significantly over-expressed in the lacrimal glands of mice subjected to desiccation stress (*P* < 0.05). The expression of TNF-α, ICAM-1, VCAM-1, and MMP-9 in the groups treated with RGN-259 2 times per day (3.3-, 2.7-, 5-, and 2.2-fold increase over the control normal group) decreased to 85%, 77.8%, 76.4%, and 81.5% at 10 days, and RGN-259 at 2 times per day (2.2-, 2.2, 2.8-, and 1.9-fold increase over the control normal group) decreased the mediators to 89.8%, 81.4%, 86.6%, and 84% at 10 days, respectively, when compared with the baseline desiccation stress-induced group (*P* < 0.05). The CsA group showed a 90.4%, 86.7%, 81.5%, and 92.3% (2.1-, 1.6-, 3.9-, and 0.9-fold increase over the control normal group) decrease in the expression of inflammatory mediators (*P* < 0.05). The LFA group showed a 92.5%, 91.1%, 90.9%, and 82.6% (1.7-, 1.1-, 1.9-, and 2-fold increase over the control normal group) decrease in the expression of inflammatory mediators, respectively, at 10 days (*P* < 0.05). No decrease was observed in the DQS group (11.5-, 13.4-, 16.3-, and 8.9-fold increase over the control normal group). The pro-inflammatory cytokines, including IFN-γ (4.3-fold increase over the control normal group), IL-1β (17.1-fold increase over the control normal group), IL-6 (25.4-fold increase over the control normal group), IL-8 (7.6-fold increase over the control normal group), and IL-17 (15.1-fold increase over the control normal group) were all up-regulated in the baseline desiccation stress-induced group (*P* < 0.05). The expression of IFN-γ, IL-1β, IL-6, IL-8, and IL-17 in the groups treated with RGN-259 at 2 times per day (4-, 3.6-, 7.5-, 1.7- and 3-fold increase over the control normal group) decreased their expression by 6.7%, 79%, 70.5%, 77.8%, and 80.1% at 10 days, and RGN-259 at 4 times per day (0.8-, 2.4, 4.9-, 0.8-, and 3.6-fold increase over the control normal group) decreased these mediators by 82.1%, 86.1%, 80.5%, 88.9%, and 76.4% at 10 days, respectively, when compared with the baseline desiccation stress-induced group (*P* < 0.05). The CsA group decreased the expression of the pro-inflammatory cytokines by 72.5%, 88.2%, 89.2%, 85.8%, and 90.4% (1.2-, 2-, 2.7-, 1.1-, and 1.5-fold increase over the control normal group, *P* < 0.05). The LFA decreased the expression of the pro-inflammatory cytokines by 90.6%, 95%, 85.4%, 88.3%, and 88.2% (0.4-, 0.9-, 3.7-, 0.9-, and 1.8-fold increase over the control normal group, *P* < 0.05). No decrease in the pro-inflammatory mediators was observed in the DQS group (6.9-, 12-, 24.3-, 5.4-, and 9.8-fold increase over the control normal group). Expression of all of the inflammatory factors in the LFA group was reduced to levels comparable to those seen in the unstressed control group. RGN-259 (both treatments) was as effective as CsA in suppressing the inflammatory factors, and administering RGN-259 at 4 times per day was more effective than giving it 2 times per day, but no significant differences were found. These findings indicate that RGN-259, CsA, and LFA were equally active for reducing inflammation whereas DQS was inactive.

### RGN-259 induced expression of mucins in both the cornea and conjunctiva

**Figure 8:**
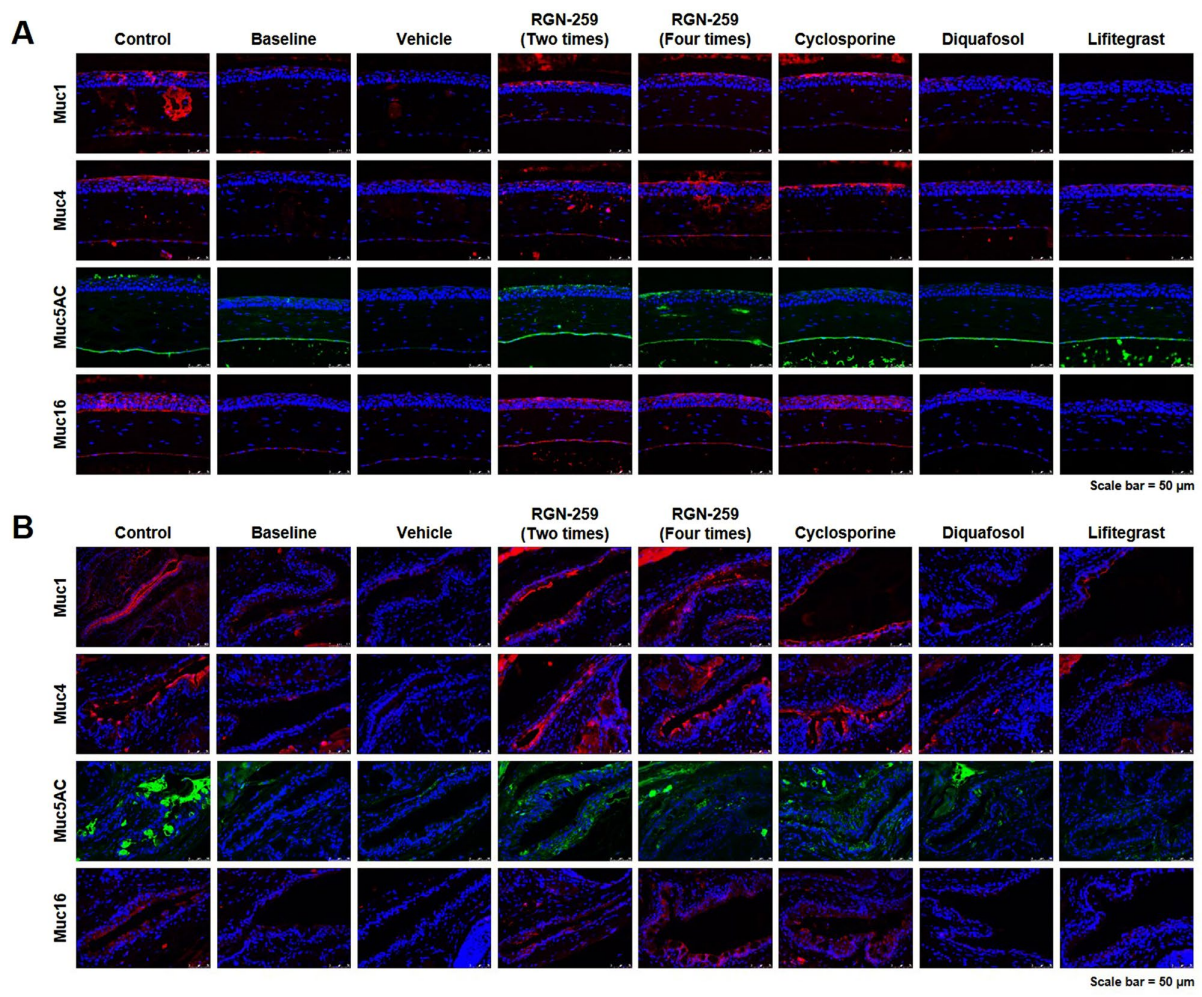
RGN-259 induced expression of mucins comparable to that of CsA. Immunofluorescence staining of mucins in the eyes of mice. (**A**) The expression of mucins in the cornea. (**B**) The expression of mucins in the conjunctiva. Scale bar = 50 μm.

Sections of the cornea (Fig. 8A) and conjunctiva (Fig. 8B) were stained by immunofluoresence for mucins, including Muc1, Muc4, Muc5AC, and Muc16. The membrane-bound mucins in the superficial layers of the corneal and conjunctival epithelia, Muc1, Muc4, and Muc16, were stained red. The baseline desiccation stress-induced group showed a decrease in the expression of these membrane-bound mucins in both the cornea and conjunctiva. The expression of Muc1, Muc4, and Muc16 were increased in both RGN-259-treated groups compared with the baseline desiccation stress-induced group. The RGN-259-treated groups showed similar increased staining of Muc1, Muc4, and Muc16 in the cornea and conjunctiva to that seen with the CsA group. The gel-forming mucin, Muc5AC, stained green in the conjunctiva. The desiccation stress-induced baseline group showed a decrease in Muc5AC expression compared with the control normal group. Compared with the baseline desiccation-stress induced group, both RGN-259 groups showed an increase in Muc5AC staining in both the cornea and conjunctiva. In both RGN-259 groups, the increased level of Muc5AC staining in the cornea was comparable to that in the CsA group. In the conjunctiva, the increase in Muc5AC staining following RGN-259 treatments was comparable to that seen in the CsA-treated group. All of the mucins showed similar changes in their expression patterns in both RGN-259-treated groups. DQS and LFA showed no increase in staining for any of the mucins and were therefore inactive in promoting mucin production after desiccation stress.

## Discussion

We evaluated the RGN-259-induced clinical and histological changes in the cornea, conjunctiva, and lacrimal glands of NOD.B10.*H2*^*b*^ mice after desiccation stress. We also compared the efficacy of RGN-259 with commercially available prescription drugs used to treat dry eye, including CsA, DQS, and LFA. Along with clinical outcomes, such as tear production, corneal irregularity, and corneal fluorescein staining, we also evaluated histological parameters, including corneal detachment, conjunctival goblet cell density, conjunctival mucin staining, and expression of inflammatory factors and mucins.

The efficacy of RGN-259 for the treatment of dry eye has been demonstrated by several clinical trials^44,45^. We observed the same improvement in our animal studies and were able to evaluate several new parameters related to reversing the signs of dry eye. Previous animal studies have been conducted with the approved dry eye drugs as detailed below. CsA, an FDA-approved dry eye treatment, has been reported to increase tear production, decrease fluorescent staining, decrease expression of inflammatory cytokines, and increase the number of goblet cells^46,47^. DQS, a topical secretagogue approved in Japan, increases mucin and tear secretion^48^. LFA, a recently approved FDA therapeutic, improves corneal fluorescence staining and has anti-inflammatory effects^49^. However, in our animal studies as detailed below, the decrease in fluorescence staining by CsA and the increase in mucin by DQS were not observed.

We assessed the efficacy of RGN-259 in a dry eye mouse model to determine the efficacy of RGN-259 relative to currently used dry eye treatment drugs. We were able to compare the efficacy of three dry eye treatment drugs in a dry eye mouse model. The efficacy of RGN-259 was compared and evaluated based on the same time point that showed the best efficacy in each parameter of the reference drugs CsA, DQS, and LFA.

In this study, mice were administered RGN-259 either 2 or 4 times per day (Fig. 1). Both RGN-259 treatments increased tear production, and the increases were comparable to those seen in the DQS- and LFA-treated groups. The improved tear production by RGN-259, DQS, and LFA was significant beginning at day 3 after instillation, but a dose-dependent effect of RGN-259 was not observed. The RGN-259, DQS, and LFA instillations seemed to stabilize the ocular surface by increasing the aqueous layer of the tear film at the early stage of tear production. Compared with the desiccation stress-induced baseline group, both RGN-259 groups showed improvement in the corneal irregularity score and in the fluorescein staining score (Figs 2 and 3). The effects on corneal smoothness and corneal fluorescein staining were significantly different in the groups receiving RGN-259 at 2 and 4 times per day over the vehicle control group. The improved corneal smoothness and fluorescein staining outcomes in both RGN-259-treated groups were comparable to those seen in the LFA-treated group, whereas CsA and DQS were inactive. RGN-259 at two and four times showed significant dose-dependent effects for both corneal smoothness and corneal fluorescein staining. The corneal smoothness improved by day 7 probably due to the stabilization of the tear film which was observed at day 3 in both RGN-259 treatment groups. The tear film was stabilized first in the RGN-259 groups, then the corneal smoothness was improved, and the damage on the ocular surface was reduced, leading to a decrease in the corneal fluorescein staining score at day 10. In a recent clinical study of RGN-259, the efficacy of RGN-259 decreased the corneal fluorescein staining score as early as day 14. It should be noted that this finding was based on a different method of scoring and different analytical methods^44,45^. Our findings suggest that RGN-259 exhibits broad clinical efficacy in the ocular surface of the dry eye model and is equally active or more active than CsA for these parameters.

Corneal epithelial cell detachment was reduced in both RGN-259 treatment groups when compared with the baseline desiccation stress group (Fig. 4). The RGN-259-associated improvement in corneal attachment was comparable to that seen in the DQS and LFA groups, while CsA was inactive for improving corneal cell attachment. RGN-259 showed significant efficacy at both doses and a trend toward a significant dose-dependent effect on the inhibition of corneal epithelial cell detachment. The effect of RGN-259 on corneal epithelial cell damage seems to be affected by the decrease in both corneal smoothness and fluorescein staining score. The conjunctival goblet cell density was significantly restored in both RGN-259 treatment groups compared with that in the baseline desiccation stressed group (Fig. 5). RGN-259 showed significant efficacy at both doses and a trend toward a significant dose-dependent effect on the number of conjunctival goblet cells. In addition, conjunctival mucin staining increased in both RGN-259 groups (Fig. 6), but the effect was not dose-dependent effect. We speculate that RGN-259 may be acting to increase goblet cell number and subsequent mucin production by the recruitment of stem cells. RGN-259 initiates tissue regeneration through recruitment of stems cells in several other injury models, including the heart, brain, and skin^50–52^. The RGN-259-associated increases in the conjunctival goblet cell number and in mucin production were comparable to that seen in the CsA group. Based on the histological appearance, DQS showed the best efficacy for mucin production in the corneal epithelial cells, and CsA exhibited the best efficacy in the conjunctival goblet cells. The findings suggest that RGN-259 has activity comparable to both CsA and DQS, and is a multi-functional agent in both the cornea and conjunctiva of the dry eye mouse model. The LFA group showed no activity for mucin production.

In patients with keratoconjunctivitis (KCS), hypersensitive and inflammatory cytokines, such as IL-17 and IFN-γ, are secreted from the resident intraepithelial lymphocytes and infiltrating CD4^+^ T cells due to desiccation in the tear film of the ocular surface^53–57^. Experimental desiccation stress increases both the number of conjunctival goblet cells positive for the Th1 cytokine IFN- γ and the concentration of IFN-γ in tears^53^. Long-term dry eye is associated with increased expression of inflammatory factors, such as TNF-α, MMP-2, MMP-9, ICAM-1, and VCAM-1, on the ocular surface^10–13^. In addition, IL-1β and TNF-α can activate the production of MMPs in epithelial cells, including MMP-9, which impairs corneal epithelial barrier function and affects membrane-associated mucin expression, on the ocular surface of the dry eye^58,59^. Patients with more severe dry eye have increased levels of IL-6 and IL-8/CXCL8 in their tears and similar increases are seen in the conjunctival epithelium in experimental dry eye models^56,60–62^. In this study, the expression of inflammatory factors was signficantly decreased in the lacrimal glands of mice treated with RGN-259 at both 2 and 4 times per day when compared with the baseline desiccation stressed group (Fig. 7). RGN-259 showed a trend toward a significant dose-dependent effect on the expression of all inflammatory factors. These decreases were comparable to those seen in the CsA- and LFA- treated mice, whereas DQS was inactive.

Patients with dry eye show a decrease in mucin expression^24^. Levels of the gel-forming mucin Muc5AC decrease in patients with Sjögren syndrome and with other types of dry eye^24,25^, as well as in several dry eye animal models^26,27^. In addition to Muc5AC, several other membrane-spanning mucins of the corneal epithelium, such as Muc1, Muc4, and Muc16, are important components of the mucous layer of the tear film^63^. In this study, mucin expression was increased in the RGN-259-treated groups, when compared with baseline desiccation stress group, and the expression of mucin staining was comparable to that seen in the CsA groups (Fig. 8). While both RGN-259 treatments significantly increased mucin expression, the effects were not dose-dependent. Both DQS and LFA had no effect on mucin production. Shirai, *et al*. reported that Muc16 affected the homeostasis of the ocular surface epithelium in mice, and this was suggested as an important concept to understand the disease mechanism underlying human dry eye patients^29^. Our results indicate that Tβ4 can normalize Muc16 expression thereby optimizing the health of the ocular surface. Although the exact mechanisms by which Tβ4 exerts these effects are not known, our study provides solid *in vivo* evidence to warrant further mechanistic studies.

Our study had the following limitations. (1) There is no comparison of dry eye drugs associated with mucin secretion. A comparative evaluation of rebamipide, a mucin secretion promoter, with RGN-259 for increasing mucin expression in both the cornea and conjunctiva of a dry eye mouse model is needed. (2) Analysis of inflammatory factors should also be done at the molecular level at various time points after instillation of the drugs. Although histological analysis of RGN-259 showed anti-inflammatory effects in the lacrimal gland of the dry eye mouse model, molecular studies of its precise mechanism are needed.

In conclusion, we found that RGN-259 treatment leads to recovery of mucins and reduction in the associated anti-inflammatory effects on the ocular surfaces in a mouse model of dry eye. RGN-259 was comparable to or even better than available prescription drugs in improving the functions of the cornea, conjunctiva, and lacrimal glands of mice.

## Methods

### Animals

The NOD.B10.*H2*^*b*^ mice were purchased from Jackson Laboratory (Bar Harbor, ME). This study was conducted in accordance with the guidelines for the care and use of laboratory animals of the Inje University College of Medicine and the Association for Research in Vision and Ophthalmology statement. All experimental protocols were approved by the Institutional Animal Care and Use Committee of Inje University College of Medicine (Approval ID: 2017-002-01).

### Experimental dry eye murine model

The experimental dry eye murine model was generated with male and female NOD.B10.*H2*^*b*^ mice that were more than 12 weeks old. Desiccation stress was achieved by a 0.5 mg/0.2 mL hypodermic injection of scopolamine hydrobromide (Sigma-Aldrich, St. Louis, MO) 4 times per day and low ambient humidity (30–40%) using an air draft from a fan for 18 hours per day for 10 days^1,7,14–16,53^. After the desiccation stress for 10 days, the mice were treated with either RGN-259, CsA, DQS, or LFA in an environment with normal humidity and temperature.

The following eight experimental groups were evaluated: control healthy group (normal, *n* = 4); baseline desiccation stressed group (untreated, desiccation stress for 10 days, *n* = 4); vehicle (*n* = 4, 5 μL/eye instilled bilaterally 4 times per day for 10 days after removal of the desiccation stress); RGN-259, 2 times (*n* = 5, 5 μL/eye instilled bilaterally 2 times per day for 10 days after removal of the desiccation stress); RGN-259, 4 times (*n* = 5, 5 μL/eye instilled bilaterally 4 times per day for 10 days after removal of the desiccation stress); CsA (*n* = 4, 5 μL/eye instilled bilaterally 2 times per day for 10 days after removal of the desiccation stress); DQS (*n* = 4, 5 μL/eye instilled bilaterally 6 times per day for 10 days after removal of the desiccation stress), and LFA (*n* = 4, 5 μL/eye instilled bilaterally 2 times per day for 10 days after removal of the desiccation stress).

### Treatments and dosing

Topical eye drops with CsA (Allergan, Irvine, CA) and LFA (Shire Pharmaceuticals, Lexington, MA) were administered twice a day. Whereas the RGN-259 eye drops (RegenTree, LLC, Princeton, NJ, USA) were administered 2 or 4 times per day. Drops containing the RGN-259 vehicle (placebo; RegenTree, LLC) were given 4 times per day. The DQS eye drops (Santen Pharmaceutical Co., Osaka, Japan) were administered 6 times per day. Ten microliters of each solution were administrated to both eyes in the mice. All measurements and evaluations were performed by three blinded observers.

### Measurement of tear production

Tear production was measured as previously described using phenol red–impregnated cotton threads (Zone-Quick; Oasis, Glendora, CA)^1,7,14–16^. The threads were placed in the lateral canthus for 20 seconds. The tear volumes were analyzed using a microscope (SZX7; Olympus Corp., Tokyo, Japan) based on the millimeters of thread that turned red due to the tears. Tear volume was measured in both eyes within 1 hour after instillation of vehicle, RGN-259, CsA, DQS, and LFA, and tear production was analyzed in both eyes, and the average value of each group was determined.

### Measurement of the corneal irregularity score

Corneal smoothness was measured using a stereoscopic zoom microscope on the ocular surface of anesthetized mice and was based on the images of the illuminated white ring observed^1,7,14–16^. The corneal irregularity scores were calculated using the following scale: 0, no distortion; 1, distortion in one quarter; 2, distortion in two quarters; 3, distortion in three quarters; 4, distortion in all four quarters; and 5, distortion so severe that no section of the ring was visible. The corneal smoothness was measured in both eyes within 1 hour after instillation of vehicle, RGN-259, CsA, DQS, and LFA, and the corneal irregularity scores was analyzed in both eyes, and the average value of each group was determined.

### Fluorescein staining in the cornea

Corneal fluorescein staining was performed as described by Rashid *et al*.^5^. A microliter of 1% sodium fluorescein was dropped onto the ocular surface of the anesthetized mice. After 1 minute, the ocular surface was washed with phosphate-buffered saline (PBS) to remove the excess fluorescein, and then corneal fluorescein staining was evaluated by photography with a cobalt blue light of a slit lamp microscope (SL-D7; Topcon, Tokyo, Japan). Corneal fluorescein staining scores were recorded using a standardized National Eye Institute grading system of 0 to 3 for each of the five areas of the cornea^6^. Corneal fluorescein staining was measured in both eyes within 1 hour after instillation of vehicle, RGN-259, CsA, DQS, and LFA, and corneal fluorescein staining scores was analyzed in both eyes, and the average value of each group was determined.

### H&E staining

Six-micrometer sections of both the eyes and adnexa were stained with H&E. Sections were de-paraffinized if necessary, hydrated in distilled water, and stained with hematoxylin (YD Diagnostics Co., Yongin, Korea) for 3 to 5 minutes followed by washing. After washing off the reaction solution with hydrochloride (HCl) solution, sections were stained with eosin (Muto Pure Chemicals Co. Ltd, Osaka, Japan) for 1 minute, and then washed again. The stained sections were dehydrated by treating sequentially with 80%, 85%, 90%, and 100% ethanol for 1 minute each. After the dehydration process, the sections were cleared by treating with both carboxylene and xylene for 1 minute and were then mounted. The sections from each group were assessed in a 0.1-mm^2^ area of either the cornea or inferior fornices of the conjunctiva. The stained sections were then examined and imaged using a virtual microscope (NanoZoomer 2.0 RS, Hamamatsu, Japan).

### Periodic Acid-Schiff (PAS) staining

Six-micrometer sections of both eyes and the adnexa were stained with a PAS kit (Merck Chemicals International, USA). The sections from each group were assessed in a 0.1-mm^2^ area of either the cornea or inferior fornices of the conjunctiva. The stained sections were then examined and imaged using a virtual microscope (NanoZoomer 2.0 RS, Hamamatsu, Japan).

### Mucin staining

Six-micrometer sections of both eyes and the adnexa were stained for mucin with Alcian blue, pH 2.5, using a kit (Abcam Inc, Cambridge, MA). The eye sections from each group were assessed in a 0.1-mm^2^ area of either the cornea or inferior fornices of the conjunctiva. The stained sections were then examined and imaged using a virtual microscope (NanoZoomer 2.0 RS, Hamamatsu, Japan).

### Immunohistochemistry

The lacrimal glands were cut into 6-µm sections using a microtome. The sections were fixed with pre-cooled acetone for 5 minutes, and then incubated with primary antibodies for TNF-α (Abcam Inc., Cambridge, MA), MMP-9 (Lifespan Biosciences Inc., Seattle, WA), ICAM-1, VCAM-1 (Bioss Inc., Woburn, MA), CD 4, IFN-γ (Novus Biologicals, Littleton, CO), IL-1β, IL-6, IL-17 (Abcam, Inc., Cambridge, UK), and IL-8 (Biorbyt, Cambridge, UK) for 1 hour at room temperature. After washing, the sections were incubated with the secondary antibody (DAKO Corp, Glostrup, Denmark) for 30 minutes. The immune reactions were visualized with diaminobenzidine chromogen, and the sections were counterstained with Mayer’s hematoxylin (Sigma) for 30 seconds at room temperature. The stained sections were photographed with a virtual microscope (NanoZoomer 2.0 RS, Hamamatsu, Japan). The calculated data were compared with the densitometry of the control group analyzed with ImageJ (National Institutes of Health, Bethesda, MD, USA).

### Immunofluorescence

The eyes and adnexa were fixed in formalin for 3 days, and then cut into 6-μm sections using a microtome. The sections were rehydrated in PBS followed by incubation in 0.3% Triton X-100 for 20 minutes. After three rinses with PBS for 5 minutes each, the sections were incubated in 3% BSA for 1 hour to block nonspecific staining. After blocking, the sections were incubated with primary antibodies, including Muc1 (1:250; Abcam Inc, Cambridge, MA), Muc4 (1:250; Bioss Inc, Woburn, MA), Muc5AC (1:250; Thermo Fisher Scientific Inc, Waltham, MA), and Muc16 (1:250; Abbiotec Inc, San Diego, CA) for 1 day. After three washes with PBS for 5 minutes each, the sections were next incubated with either fluorescein isothiocyanate (FITC)-conjugated donkey anti-mouse immunoglobulin G (IgG), or donkey anti-rabbit (1:500; Thermo Fisher Scientific Inc, Waltham, MA) secondary antibody for 1 hour. The stained sections were washed in 3 times (5 minutes each) with PBS and then counter stained and mounted with mounting medium containing 4,6-diamino-2-phenylindole (DAPI; Southern Biotech Inc, Birmingham, AL). The sections were observed under a fluorescence microscope (Leica DM2500, Leica Microsystems GmbH, Wetzlar, Germany).

### Statistical analysis

The data were analyzed using GraphPad Prism version 5.0 (GraphPad Prism, San Diego, CA) for Windows and expressed as a mean ± standard deviation (SD). The differences between the groups were analyzed using a one-way analysis of variance (analyses of variance with the Tukey’s test), and statistical significance was defined as *P* < 0.05.
